# Autophagy Gene Panel-Based Prognostic Model in Myelodysplastic Syndrome

**DOI:** 10.3389/fonc.2020.606928

**Published:** 2021-02-05

**Authors:** Ming-Jing Wang, Wei-Yi Liu, Xue-Ying Wang, Yu-Meng Li, Hai-Yan Xiao, Ri-Cheng Quan, Gang Huang, Xiao-Mei Hu

**Affiliations:** ^1^ Department of Hematology, Xiyuan Hospital, China Academy of Chinese Medical Sciences, Beijing, China; ^2^ Graduate School, China Academy of Chinese Medical Sciences, Beijing, China; ^3^ Graduate School, Beijing University of Chinese Medicine, Beijing, China; ^4^ Divisions of Pathology and Experimental Hematology and Cancer Biology, Cincinnati Children’s Hospital Medical Center, Cincinnati, OH, United States

**Keywords:** myelodysplastic syndrome, autophagy, prognostic model, myelodysplastic syndrome, autophagy-related genes

## Abstract

Abnormal autophagy is related to the pathogenesis and clinical symptoms of myelodysplastic syndrome (MDS). However, the effect of autophagy-related genes (ARGs) on the prognosis of MDS remains unclear. Here, we examined the expression profile of 108 patients with MDS from the GSE58831 dataset, and identified 22 genes that were significantly associated with overall survival. Among them, seven ARGs were screened and APIs were calculated for all samples based on the expression of the seven ARGs, and then, MDS patients were categorized into high- and low-risk groups based on the median APIs. The overall survival of patients with high-risk scores based on these seven ARGs was shorter than patients with low-risk scores in both the training cohort (P = 2.851e-06) and the validation cohort (P = 9.265e-03). Additionally, API showed an independent prognostic indicator for survival in the training samples [hazard ratio (HR) = 1.322, 95% confidence interval (CI): 1.158–1.51; P < 0.001] and the validation cohort (HR = 1.05, 95% CI: 1–1.1; P < 0.01). The area under the receiver operating characteristic curve (AUROC) of API and IPSS were 43.0137 and 66.0274 in the training cohorts and the AUC of the validation cohorts were 41.5361 and 72.0219. Our data indicate these seven ARGs can predict prognosis in patients with MDS and could guide individualized treatment.

## Introduction

Myelodysplastic syndrome (MDS) is a malignant clonal hematopoietic stem cell disorder characterized by the proliferation of bone marrow primordial cells and a decrease in peripheral blood cells (1). About a third of MDS patients will develop acute myeloid leukemia (2, 3). MDS was found to be related to genetic mutations or epigenetic modifications, which lead to abnormal autophagy, apoptosis of mature cells, chromosomal abnormalities, and a high level of inflammation in the bone marrow microenvironment (4–8). Therefore, targeting these processes that are involved in the pathogenesis of MDS may improve patient outcomes.

The prognosis of patients with MDS is currently assessed using either the World Health Organization (WHO) classification-based Prognostic Scoring System (WPSS) (9), the International Prognostic Scoring System (IPSS) (10), MD Anderson risk model score for MDS (MDACC) (11), or the Revised International Prognostic Scoring System (IPSS-R) (12). Nowadays, the IPSS-R (which is based on peripheral blood cell counts, marrow blast percentage, and cytogenetics) is most widely used for assessing patients with MDS (12). Although the utility of these prognostic assessment systems has been confirmed in clinical practice, they do not take gene mutations into account (13). However, with the development of gene expression profile and new high-throughput technology, the understanding of the pathogenesis of MDS is getting further and better. Multiple gene mutations have been identified and considered as important substrates for the development of MDS, such as RNA splicing, histone manipulation, DNA methylation, transcription factors, kinase signaling, DNA repair, cohesin proteins, and other signal transduction elements. These findings also have a great influence on the judgement of prognostic, the selection of therapies, and future treatment endeavors.

Therefore, high-risk patients may be inadequately treated, and low-risk patients may be over-treated based on the present prognostic assessment systems. As such, a more comprehensive and diverse prognostic assessment system for patients with MDS is required (14). Autophagy is a catabolic process involved in cellular defense and the stress response (15) and plays an essential role in the differentiation of hematopoietic cells. The disorder of autophagy mechanisms resulting in BM microenvironment changes and hematopoiesis obstruct, and multiple studies have shown that abnormal autophagy is related to the pathogenesis and clinical symptoms of MDS (16–18). However, these studies have focused on the effect of a single autophagy gene or a minority of autophagy genes on MDS. Therefore, the relationship between MDS and multiple autophagy-related genes (ARGs) remains unclear. This study aimed to develop a new prognostic model for MDS based on the expression of multiple ARGs related to clinical characteristics.

## Materials and Methods

### Data Collection

The mRNA expression profiles and relevant clinical information for the training (GSE58831 (19)) and validation (GSE114922 (20)) cohorts were downloaded from the GEO database. All expression files were normalized and log2 transformed. The analysis of differentially expressed (DE) genes was performed using the Wilcoxon Test, and the *P* < 0.05 was considered a significant category. All 232 ARGs were obtained from the Human Autophagy Database (HADB, http://autophagy.lu/clustering/index.html). Mutation variants of the ARGs were identified using the cBioportal for Cancer Genomics database (21, 22) (http://www.cbioportal.org/).

### Functional Analysis

The R studio software (https://rstudio.com/) was used to perform the GO enrichment and KEGG functional analysis. A *P* < 0.05 was considered a significant category.

### Co-Expression Analysis

Co-expression analysis was performed by string tools (http://string-db.org/cgi/input.pl).

### Construction of Prognostic Model Based on ARGs

Prognosis-related genes were distinguished using a multivariate cox regression model. After integrating the expression values for each gene, a risk scoring formula was computed for each patient and weighted by its estimated regression coefficients. The risk scores were generated for each patient using this formula in the training cohort and validation cohort. Then, the patients were categorized into a low-risk group and a high-risk group based on the median risk score. Survival differences between the two groups were analyzed by the Kaplan-Meier method and compared using log-rank statistical methods. Univariate analysis and multivariate cox regression analysis and stratified analysis were performed to test and verify the independence of risk scores in predicting patient outcomes. Receiver operating characteristic (ROC) curves were used to evaluate the accuracy of model predictions. The specific steps used to develop the model for predicting prognosis are shown in **Supplementary Figure 1**.

## Results

### Patient Characteristics

RNA-seq and clinical data of 108 MDS samples from the GSE58831 dataset were used to construct the ARGs-MDS prognostic model. In this dataset, there are 53 patients with high-risk, consisting of 39 male and 14 female, and the age of 41 patients were above 60 years old, while 12 patients were below 60. The other 55 patients were low-risk according to autophagy prognostic index (API), which containing 32 male and 23 female, and the age of 37 patients were above 60 years old, while 18 patients were below 60. Additionally, data of 80 MDS samples from the GSE114922 dataset served as the validation cohort. In the GSE114922 dataset, there are 41 patients with high-risk, consisting of 28 male and 13 female, and the age of 33 patients were above 60 years old, while 8 patients were below 60. The other 39 patients were low-risk according to API, which containing 19 male and 20 female, and the age of 28 patients were above 60 years old, while 11 patients were below 60. The detailed characteristics of the patients including WHO category, karyotype (IPSS), and IPSS are shown in **Table 1**.

**Table 1:** The detailed patient characteristics of training cohort and external validation cohort in MDS.

Characteristics	Training cohort		P‐ value	Validating cohort		P ‐value
Risk	High risk	Low risk		High risk	Low risk	
Patients	53	55		41	39	
Gender						
Male	39	32	0.1	28	19	0.1
Female	14	23		13	20	
Age						
≥61	41	37	0.2	33	28	0.4
≤60	12	18		8	11	
WHO_category						
MDS-U	3	1	0.91	/	/	0.26
EB1/EB2	25	15		13	3	
RS-SLD/MLD	1	14		11	3	
SLD/MLD	23	20		16	34	
5q‐	3	3		/	/	
Karyotype_IPSS						
Bad	11	8	0.81	26	23	0.37
Intermediate	25	21		12	12	
Good	17	26		3	4	
IPSS						
High	5	1	0.12	3	/	0.08
Int‐1	23	26		20	16	
Int‐2	12	8		10	3	
Low	13	20		8	20	

### Identification of Differentially Expressed ARGs

**Figure 1:**
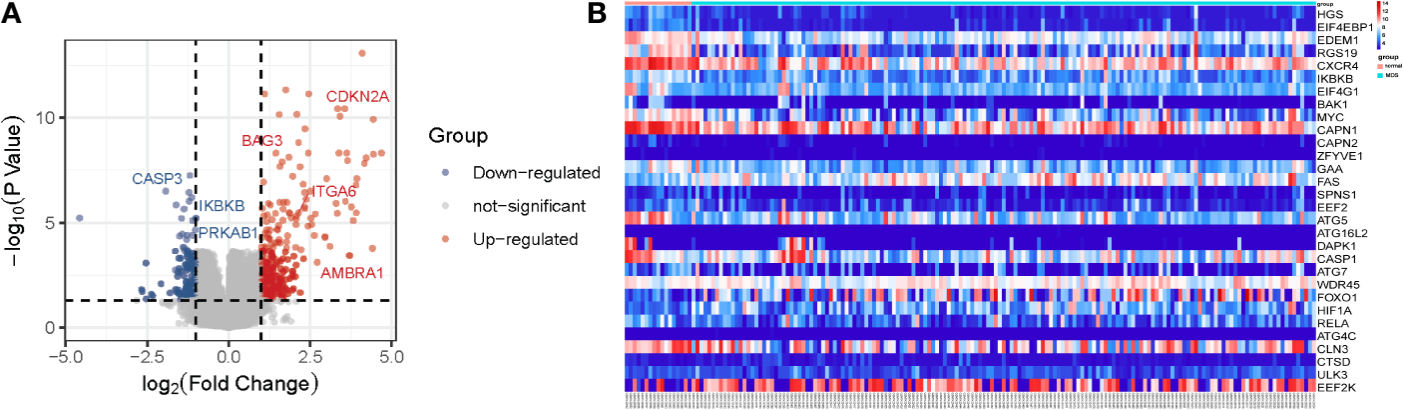
Differentially expressed ARGs in MDS. Volcano map **(A)** showing all differentially expressed genes between patients with MDS and healthy subjects. Significantly upregulated genes are shown as red dots, significantly downregulated genes as green dots, and genes showing no difference in expression are shown as grey dots. Heatmap **(B)** showing the expression of top 30 ARGs in MDS and normal samples.

In order to screen the differentially expressed ARGs, we first carried out the differential gene analysis by limma package of R tools. In total, 315 differential genes were identified for further functional enrichment analysis based on the criteria of P < 0.05 and |logFC|≥1 (**Figure 1A**). After that, 93 differentially expressed ARGs of 232 ARGs from HADB database were extracted from 315 differential genes in GSE58831 dataset for further analysis (**Supplementary Figure 2**). Here, we present the top 30 genes of 93 differentially expressed ARGs. The results of 30 differentially expressed ARGs between MDS and normal samples were visualized as heatmap in **Figure 1B**. Among these 30 ARGs, 8 ARGs were up-regulated (FAS, ATG16L2, WDR45, FOXO1, HIF1A, ATG4C, CTSD, and EEF2K) and 22 were down-regulated (HGS, EIF4EBP1, EDEM1, RGS19, CXCR4, IKBKB, EIF4G1, BAK1, MYC, CAPN1, CAPN2, ZFYVE1, GAA, SPNS1, EEF2, ATG5, DAPK1, CASP1, ATG7, RELA, CLN3, and ULK3) in patients with MDS (**Figure 1B**).

### Functional Enrichment of Differentially Expressed Genes

**Figure 2:**
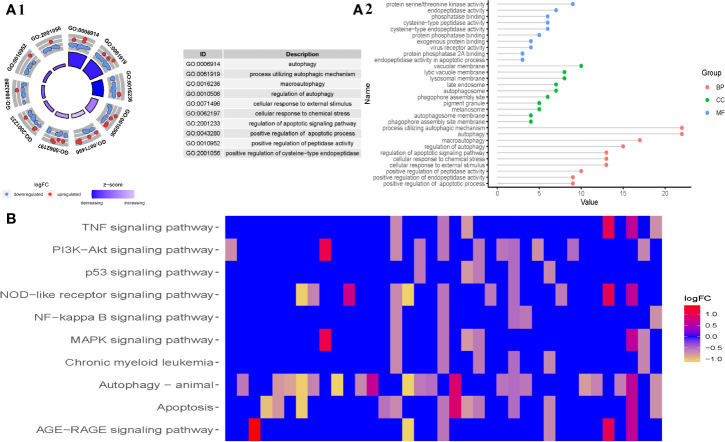
Gene functional enrichment analysis of differentially expressed ARGs in MDS. **(A1)** The top 10 results of the GO enrichment analysis of differentially expressed ARGs, and **(A2)** the top 10 for each category (biological processes, molecular functions, and cellular components). **(B)** Top 10 of KEGG pathway analysis of differentially expressed ARGs.

To better realize the role of differentially expressed genes (DEG) in MDS, we then performed functional enrichment analysis of the 315 differential expressed genes. The top 10 results of the GO and KEGG enrichment are summarized in **Figures 2A, B**, respectively. GO enrichment contained three parts: biological process (BP), cellular component (CC), and molecular function (MF). As the results showed in **Figure 2A**, in the BP parts, the DEG were mainly enriched in autophagy, process utilizing autophagic mechanism, macroautophagy, regulation of autophagy, cellular response to external stimulus, cellular response to chemical stress, regulation of apoptotic signaling pathway, positive regulation of apoptotic process, positive regulation of peptidase activity, positive regulation of endopeptidase activity, and so on. In the CC parts, the DEGs were mainly enriched in phagophore assembly site, autophagosome, phagophore assembly site membrane, vacuolar membrane, autophagosome membrane, lysosomal membrane, lytic vacuole membrane, late endosome, melanosome, pigment granule, and so on. In the MF parts, the DEGs were mainly enriched in cysteine-type endopeptidase activity, protein serine/threonine kinase activity, endopeptidase activity in apoptotic process, cysteine-type peptidase activity, phosphatase binding, protein phosphatase binding, virus receptor activity, exogenous protein binding, protein phosphatase 2A binding, endopeptidase activity, and so on.

All in all, GO enrichment showed the differential expressed genes were mainly involved in autophagy, apoptosis, and endopeptidase regulation. The z scores of these GO enrichment analysis were >0, indicating that the DEGs were upregulated in these BP, CC, and MF, while the z scores of these GO enrichment analysis were <0 suggested that the DEGs were downregulated in these BP, CC, and MF.

In addition, KEGG enrichment showed that the DEGs in MDS are primarily involved in the TNF signaling pathway, PI3K-Akt signaling pathway, p53 signaling pathway, NOD-like receptor signaling pathway, NF-kappa B signaling pathway, MAPK signaling pathway, Chronic myeloid leukemia, autophagy-animal signaling pathway, apoptosis signaling pathway, and AGE-RAGE signaling pathway, among others.

### Establishment of the Prognostic Model Based on ARGs

**Figure 3:**
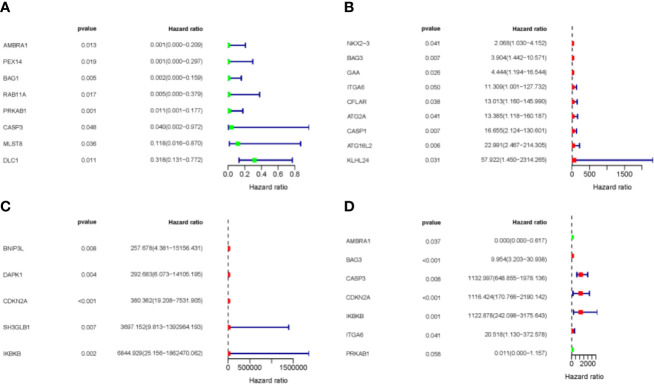
Expression profile and prognostic value of ARGs. **(A–C)** Univariate regression analysis. **(D)** Multivariate regression analysis. Red represents positively correlated with prognostic, and green represents negatively correlated prognostic.

To analyze the prognostic value of the ARGs in MDS progression, first we performed univariate analysis to screen for ARGs related to prognosis from 93 differentially expressed ARGs. A total of 22 genes from the GSE58831 dataset were identified, eight of which were negatively correlated with survival, and 14 were positively correlated (**Figures 3A–C**). Seven of these 22 genes were significantly associated with prognosis after multivariate analysis (**Figure 3D**). The expression patterns of these seven genes are shown in **Supplementary Figure 3**.

**Figure 4:**
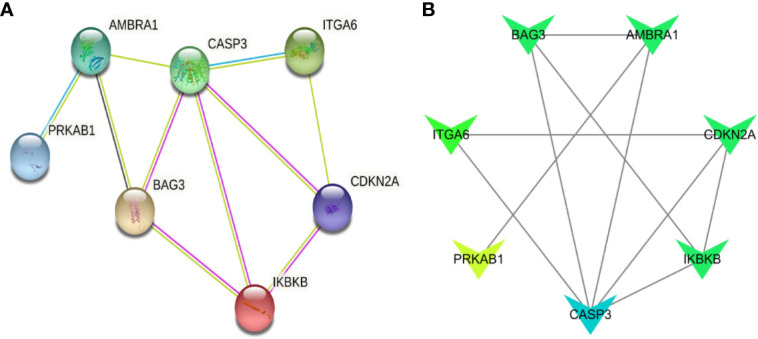
Co-expression analysis of seven ARGs. **(A)** The co-expression network of seven ARGs. **(B)** The degree of association between the seven genes, the darker the color, the more connections.

In order to figure out the relationship of the seven significant genes, co-expression analysis were performed. Then, the results of co-expression analysis of these seven genes showed that these seven key ARGs have a regulatory relationship with each other. In this co-expression analysis network, CASP3 was dominant because it has the most associations. (**Figures 4A, B**). Considering the clinical significance of these ARGs, we also looked up their genetic mutation information in MDS patients. Although the mutation rates of these seven genes are not notable, they still have significant prognostic value (**Supplementary Figure 4**). Thus, the information of the genetic mutations put an emphasis on the importance of these seven ARGs in MDS.

Based on the results of the multivariate cox regression analysis, we constructed an API to divide MDS patients into two groups according to median of risk score. The API was calculated as follows:

$$\begin{array}{c} \text{Risk score}=[\text{Expression level of AMBRA}1*(-8.26839)]+[\text{Expression}\\ \text{level of BAG}3*(2.29799)]+[\text{Expression level of CASP}3*(7.03262)]+\\ {}[\text{Expression level of CDKN}2\text{A}*(8.71873)]+[\text{Expression level of IKBKB}*\\ (13.68406)]+[\text{Expression level of ITGA}6*(3.02128)]+[\text{Expression level of}\\ \text{PRKAB}1*(-4.54978)] \end{array}$$

### The Relevance of ARGs and OS in MDS Patients

**Figure 5:**
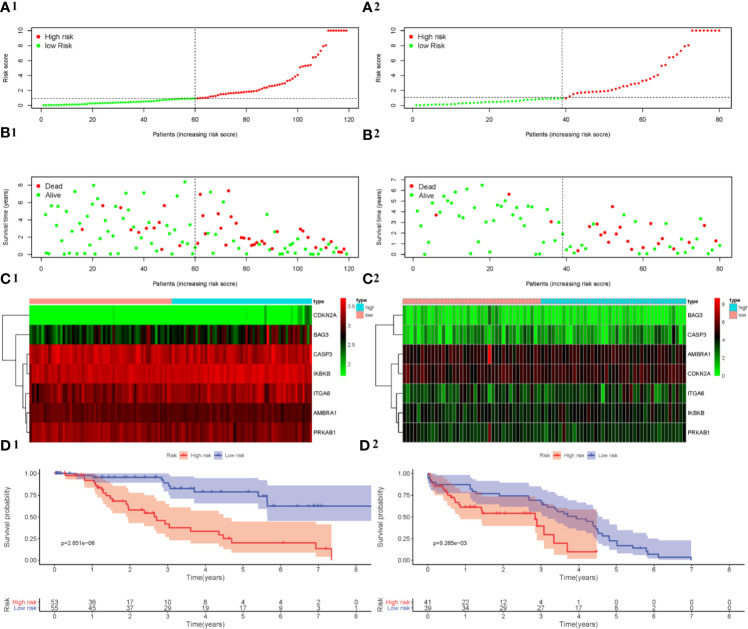
Development and verification of a prognostic index based on ARGs. The distribution of the prognostic index in the training cohort is shown in **(A1)** and the validation cohort is shown in **(A2)**. The survival status of patients in the training cohort **(B1)** and the validation cohort **(B2)**. The expression profile of ARGs in the training cohort **(C1)** and the validation cohort **(C2)**. Kaplan-Meier survival curves for the training cohort **(D1)** and the validation cohort **(D2)**.

In order to figure out the ability of the API for OS prediction, Kaplan-Meier analysis was performed to evaluate the OS outcomes in the high-risk group and low-risk group. The risk score of patients in the high-and low-risk groups from the training cohort (GSE58831) were visualized in **Figure 5A1**. As the risk score increased, a rising number of patients died (**Figure 5B1**). The **Figure 5C1** showed the expression of the seven ARGs in the two groups. Using this API, we also showed survival was significantly poorer in patients from the high-risk group than those from the low-risk group in the training cohort (*P* = 2.851e-06, **Figure 5D1**). Patients in the validation cohort (GSE11 4922) were also divided into low- and high-risk groups using the same API calculation formula from the training cohort. As for the results of Kaplan-Meier analysis in validation cohort, patients from the high-risk group also had a poorer outcome (**Figures 5A2–D2**). These results showed that the risk score accurately reflects the survival of patients and that the autophagy-related signature for OS accurately predicts the prognosis of patients.

### Independent Prognostic Analysis

**Figure 6:**
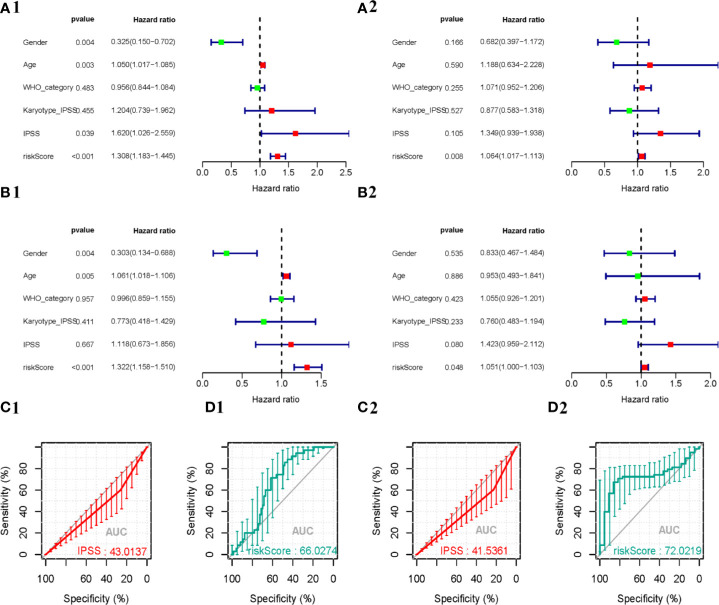
Predictive performance of prognostic indicators based on ARGs. The result of Cox regression analysis in MDS are shown as forest plots, including univariate analysis outcomes in the training **(A1)** and validation **(A2)** cohorts, and multivariate analysis outcomes in the training **(B1)** and validation **(B2)** cohorts. Survival-dependent receiver operating characteristic (ROC) curves validate the prognostic significance of IPSS in the training **(C1)** and validation **(C2)** cohorts and ARGs-based prognostic indicators in the training **(D1)** and validation **(D2)** cohorts.

To determine whether the autophagy-related signature for OS is an independent prognostic factor for MDS patients, univariate COX analysis and multivariate COX regression analysis were performed. Univariate analysis showed that the API was significantly associated with patient prognosis (**Figures 6A1, A2**). In addition, after adjusting for clinicopathological features (such as age, gender, IPSS, WHO-category, and Karyotype-IPSS), API remained an independent prognostic indicator for survival in the training samples [hazard ratio (HR) = 1.322, 95% confidence interval (CI) = 1.158–1.51; P < 0.001; **Figure 6B1**] and the validation samples (HR = 1.051, 95% CI = 1–1.1; P < 0.05; **Figure 6B2**) in our multivariate analysis. Then, a ROC curve was constructed to determine the predictive accuracy of the autophagy-related signature. Moreover, the area under the receiver operating characteristic (AUC) curve of IPSS and API were 43.0137 and 66.0274 in the training cohorts (**Figures 6C1, 6C2**) and the AUC of the validation cohorts were 41.5361 and 72.0219 (**Figures 6D1, 6D2**), respectively, which indicated a better predictive accuracy of API. Together these data indicate that the API can predict survival in patients with MDS.

### Clinical Utility of the Prognostic Signature

**Figure 7:**
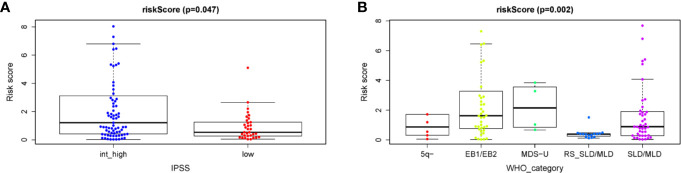
Clinicopathological significance of the prognostic index based on the 7-ARG signature in MDS patients compared to **(A)** the IPSS score and **(B)** the WHO category.

Finally, in order to realize whether the autophagy-related prognostic signature for OS affects the progression of MDS, the relationship between the API and the clinicopathological variables of patients with MDS were analyzed. We found a significant correlation between API and Clinical classification WHO prognostic system according to the WHO prognostic system (P = 0.002, **Figure 7B**). We also noticed that API was significantly correlated with IPSS (P = 0.047, **Figure 7A**), and the API risk score of int/high risk patients was higher than these in low risk patients. However, these results also indicate that the API and IPSS make approximately the same judgments about patient prognosis, which helps to demonstrate the reliability of the API. Thus, the prognostic signature for OS could accurately predict the progression of MDS.

## Discussion

MDS is still one of malignant diseases of human blood system, which is characterized by ineffective hematopoiesis of the bone marrow, long-term progressively refractory anemia, and frequent development of leukemia (23). The incidence of this disease is 3 ≤ 4/10^5^ (24). Morbidity increasing along with the age, and among people over 60 years old, the incidence rate is about 7 ≤ 35/10^5^. In addition, the incidence of female is higher than male (25). In some patients, the cause of cytopenia(s) is uncertain, even after thorough clinical and laboratory evaluation (26). Actually, it can be occurred at any age, and patients always have a long course of disease with huge differences in prognosis. Nevertheless, anappropriate risk stratification is necessary for prognosis judgment and the development of treatment strategy. Currently, IPSS-R is the most commonly used tool for risk stratification in MDS. This score system stratifies patients into five groups (very low, low, moderate, high, very high) according to the severity of cytopenia, the percentage of bone marrow blasts, and the specific kind of cytogenetic abnormalities. In clinical practice, it is helpful to forecast the prognosis of MDS, but it continues to have limitations: molecular information is meaningful, but lacking; the differences of OS in low risk group and very low risk group are not notable; the reproducibility and stability of OS in moderate risk group is inconsistent (27–29). As for the other widely used prognosis evaluation model (WPSS, IPSS, and MDACC), they have limitations too.

IPSS was proposed to evaluate the survival rate and the risk of leukemia transformation based on the percentage of primordial cells, chromosome karyotype and peripheral blood cell reduction (10). The IPSS system has become one of the most commonly used prognostic scoring systems for its strong applicability, but IPSS ignores the factors that are closely related to the prognosis of patients, such as the dependence of red blood cell transfusion, severe hemocytopenia, cell dysplasia, and chromosome karyotype (related to bone marrow primordial cells) (30–32).

WPSS was established based on WHO classification, which was considered to be making good use of the prognosis ability of WHO classification and overcome the shortcomings of IPSS scoring system. The system replaces the item of decrease of blood cells in IPSS scoring system with the dependence of red blood cell infusion, but still retains the cytogenetic prognosis group in IPSS scoring system. This change made WPSS can be used at any stages of disease rather than just at the initial diagnosis stage (30). However, the item of the dependence of red blood cell transfusion in WPSs scoring system has always been controversial because it is susceptible to subjective factors (33). Finally, this item was replaced by anemia degree, but WPSS is still failed to evaluating t-MDS, CMML and MDS/MPN overlapping phenotypes, just like IPSS and IPSS-R (9). Since the above limitations, researchers developed MDACC scoring system (11). This scoring system can give evaluation at any time and any stage of MDS patients without reference to WHO classification, but its value still needs to be further study in clinical practice.

Recently, multiple studies suggest the pathogenesis of MDS is driven by an abnormality in autophagy. For example, low expression of AGT7 (34) and other autophagy regulation defects (35) have been observed in MDS. Moreover, the expression of the autophagy-associated marker LC3B is positively correlated with hemoglobin levels, indicating that autophagy might be involved in MDS-associated anemia (16). Furthermore, some ARGs have been associated with therapeutic response and prognosis of MDS (36, 37). Despite the emerging evidence, an autophagy-related model for predicting prognosis in patients with MDS has not been proposed. In the present study, we constructed an API for stratifying patients with MDS. First of all, differential expression analysis between MDS patients and normal marrow were carried out, and then, DEGs were obtained. After that, GO and KEGG analyses based on the DEGs were performed to realize the relationship of autophagy and MDS. Then, the results of GO functional analysis showed that these DEGs were mainly enriched in autophagy, apoptosis, and endopeptidase regulation. KEGG enrichment showed that the DEGs in MDS are also closely related to the autophagy signaling pathways, apoptosis signaling pathways, et al. The results of GO and KEGG analysis indicated that ARGs were related to MDS, which consistent with the former studies (34, 35). However, further experiments are still needed to verify the role of autophagy in MDS. To determine whether the prognostic value of the ARGs in MDS progression, we performed univariate analysis to screen for ARGs related to prognosis. A total of 22 genes were identified. We also carried out multivariate cox analysis to make sure the prognostic value of the 22 genes. Then, 7 of these 22 genes were significantly associated with prognosis after multivariate analysis. According to the expression of these seven ARGs, we build API formula and calculated API scores for all patients. Then, MDS patients can be classified into high risk score group and low risk score group by the API. Moreover, the survival of MDS was significantly higher in the low risk score group than in the high risk score group. These results indicate that APIs, which were based on the expression of seven ARGs, have good prognostic value. We also noticed that these seven genes were co-expressed and CASP3 was dominant, which suggested that CASP3 has the potential to become a new treatment target associated with MDS prognosis. Moreover, the information of the genetic mutations put an emphasis on the importance of these seven ARGs in MDS. Finally, we identified seven ARGs for prognostic stratification in patients with MDS from GSE58831dataset. Based on these seven ARGs, API was constructed for stratifying patients with MDS. Additionally, this API also has been further validated in the cohort from GSE114922 dataset. In terms of the clinical relevance of the API, we noticed that API was significantly correlated with IPSS. This finding shows a good uniformity and complementarity between the IPSS and the API.

We also examined the role of seven ARGs in tumors. The seven genes identified in our study have previously been connected to the prognosis of myeloid malignancy and other tumors. In particular, CDKN2A is overexpressed in bone marrow mesenchymal stromal cells (BM-MSCs) in patients with MDS, and CDKN2A knockdown can promote the proliferation of BM-MSCs (38). In addition, ITGA6 (CD49f) regulates the differentiation, adhesion, and migration of BM-MSCs, and may promote inflammation in the bone marrow microenvironment (39). Similarly, IKBKB is related to inflammation and infection, and a persistent inflammatory response is a potential cause of tumors (40). Meanwhile, PRKAB1 (AMPK) is essential for the differentiation of hematopoietic cells and is a potential target for MDS treatment (41). CASP3 plays a critical role in apoptosis and is involved in the occurrence and development of malignant tumors (42). BAG3 appears to maintain tumor growth and regulate metastasis (43). Finally, AMBRA1 is a target of mTOR, which promotes dephosphorylation and regulates cell proliferation (44). However, despite the proposed functions of these seven ARGs in various types of cancer, their role(s) in the pathogenesis of MDS should be further investigated.

However, there are some limitations to this study. First, due to the limited sample size, there may be some unavoidable bias. Second, to determine the robustness of the API, further validation in other independent cohorts is necessary. Third, the relationship of the ARGs in our model to the pathogenesis of MDS requires further verification in functional experiments and clinical practice.

In summary, we built a prognostic model for MDS based on a comprehensive analysis of the expression profiles of ARGs and related clinical features from a GEO dataset. We validated the model in an independent dataset, and found good uniformity in the training and verification sets. This new risk scoring model can help assess the prognosis of MDS patients, but further experiments are still needed to verify our findings in the future.

## Data Availability Statement

The datasets presented in this study can be found in online repositories. The names of the repository/repositories and accession number(s) can be found in the article/**Supplementary Material**.

## Author Contributions

X-MH designed the study. M-JW and W-YL analyzed the data and wrote the manuscript. X-YW and Y-ML made graphics. H-YX and R-CQ participated in design. GH and X-MH checked the final manuscript. All authors contributed to the article and approved the submitted version.

## Funding

This study was supported by a Grant from the National Natural Science Foundation of China (grant no. 81673821) to X-MH, a Grant from the National Natural Science Foundation of China (grant no. 81774142) to H-YX, and a Grant from the Special Research Foundation of Central Level Public Scientific Research Institutes (grant no. ZZ10-016) to X-MH.

## Conflict of Interest

The authors declare that the research was conducted in the absence of any commercial or financial relationships that could be construed as a potential conflict of interest.

## Supplementary Material

Supplementary Figure 1 Flowchart showing the development of a prognostic model for MDS based on ARGs.

Supplementary Figure 2 The expression patterns of the ARGs in MDS. 7 prognosis-related ARGs in MDS.

Supplementary Figure 3 The expression patterns of the ARGs in MDS. 93 different expression of ARGs in MDS.

Supplementary Figure 4 Mutations in prognosis-related ARGs. This dataset was obtained from cBioportal for Cancer Genomics database, which containing 4,396 MDS samples. The mutation rate of 7 prognosis-related ARGs after multivariate analysis and 22 prognosis-related after univariate analysis were showed in **(A**, **C)**. The survival analysis of 7 prognosis-related ARGs were showed in **(B)**.
