# Supplementary figures and images for: Autophagy Gene Panel-Based Prognostic Model in Myelodysplastic Syndrome
Source: Front Oncol. 2021 Feb 5;10:606928. doi: 10.3389/fonc.2020.606928 (PMC7894207; doi:10.3389/fonc.2020.606928)

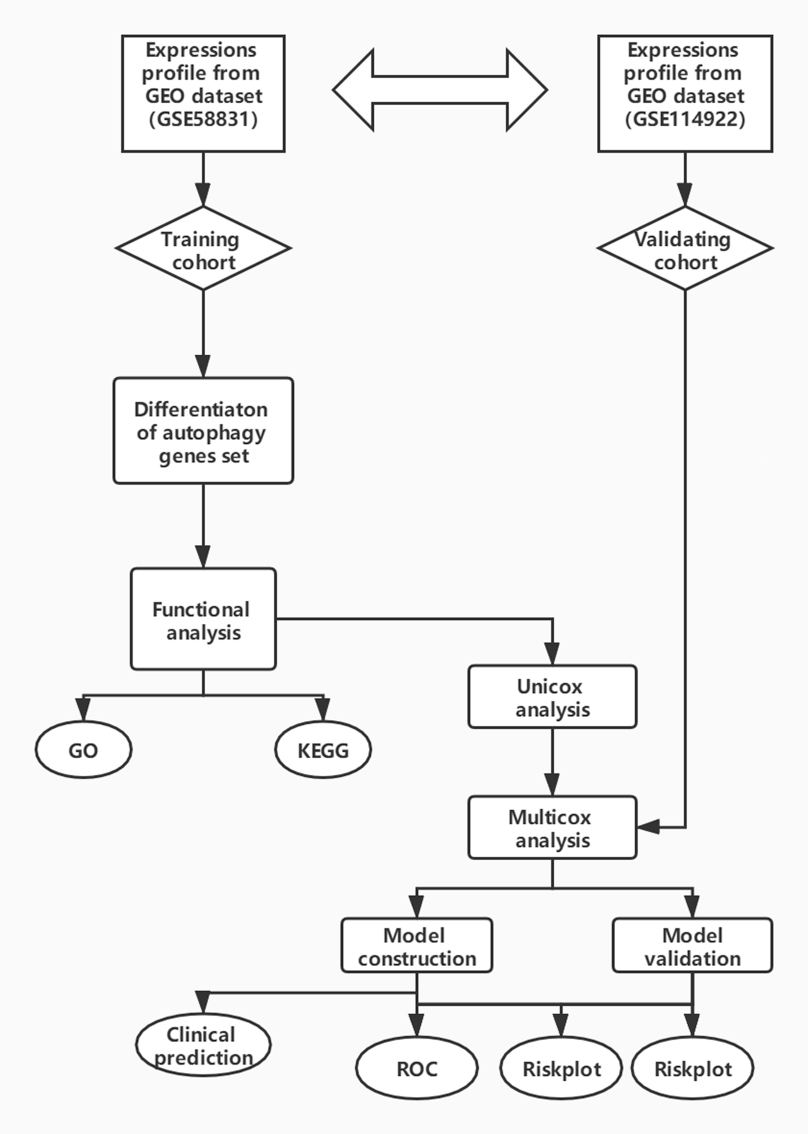

Supplement: Supplementary Figure 1 — Flowchart showing the development of a prognostic model for MDS based on ARGs. [file Image_1.tif]

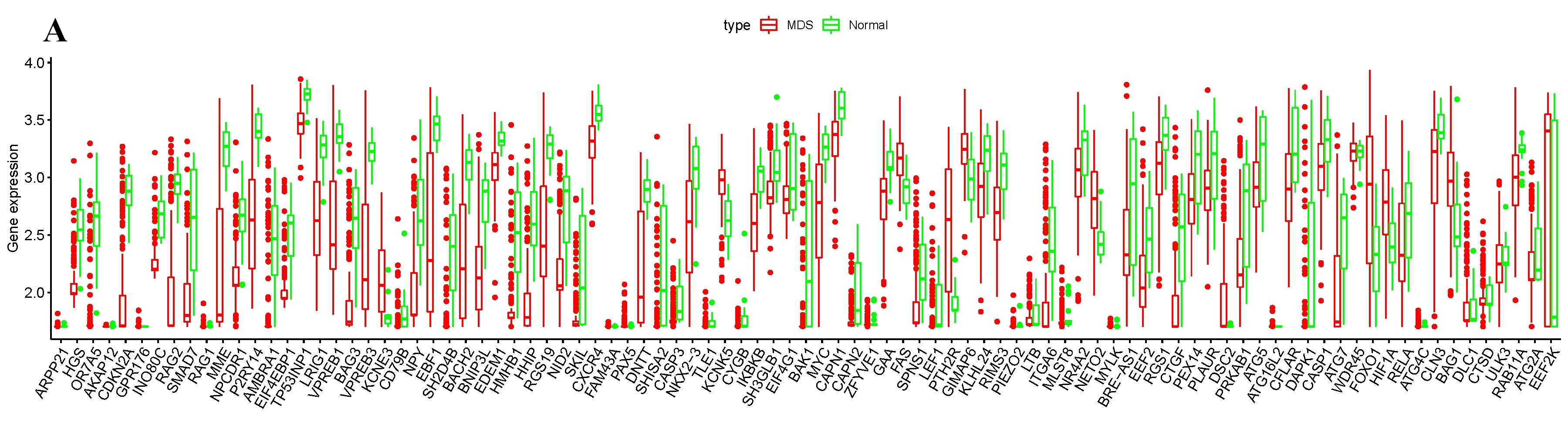

Supplement: Supplementary Figure 2 — The expression patterns of the ARGs in MDS. 7 prognosis-related ARGs in MDS. [file Image_2.jpeg]

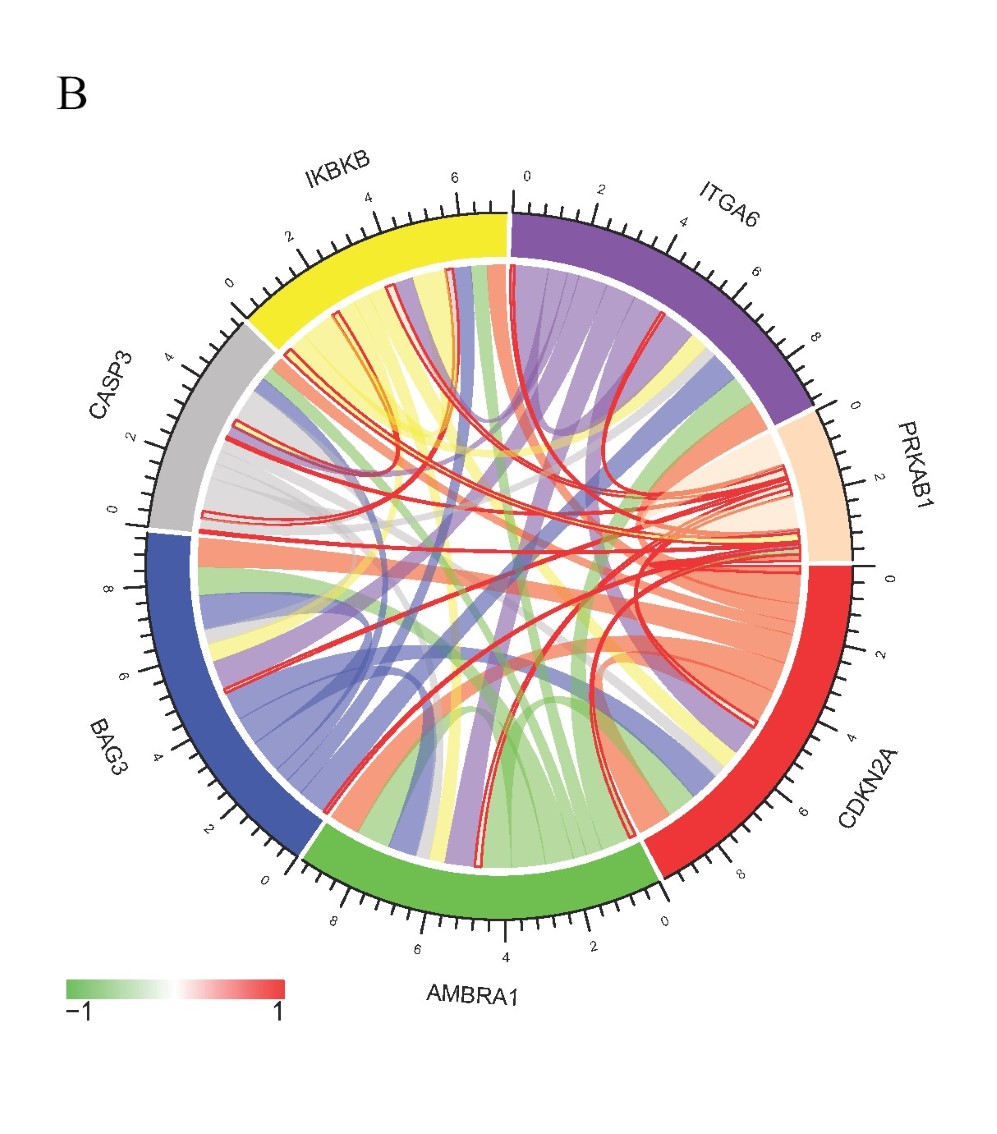

Supplement: Supplementary Figure 3 — The expression patterns of the ARGs in MDS. 93 different expression of ARGs in MDS. [file Image_3.jpeg]

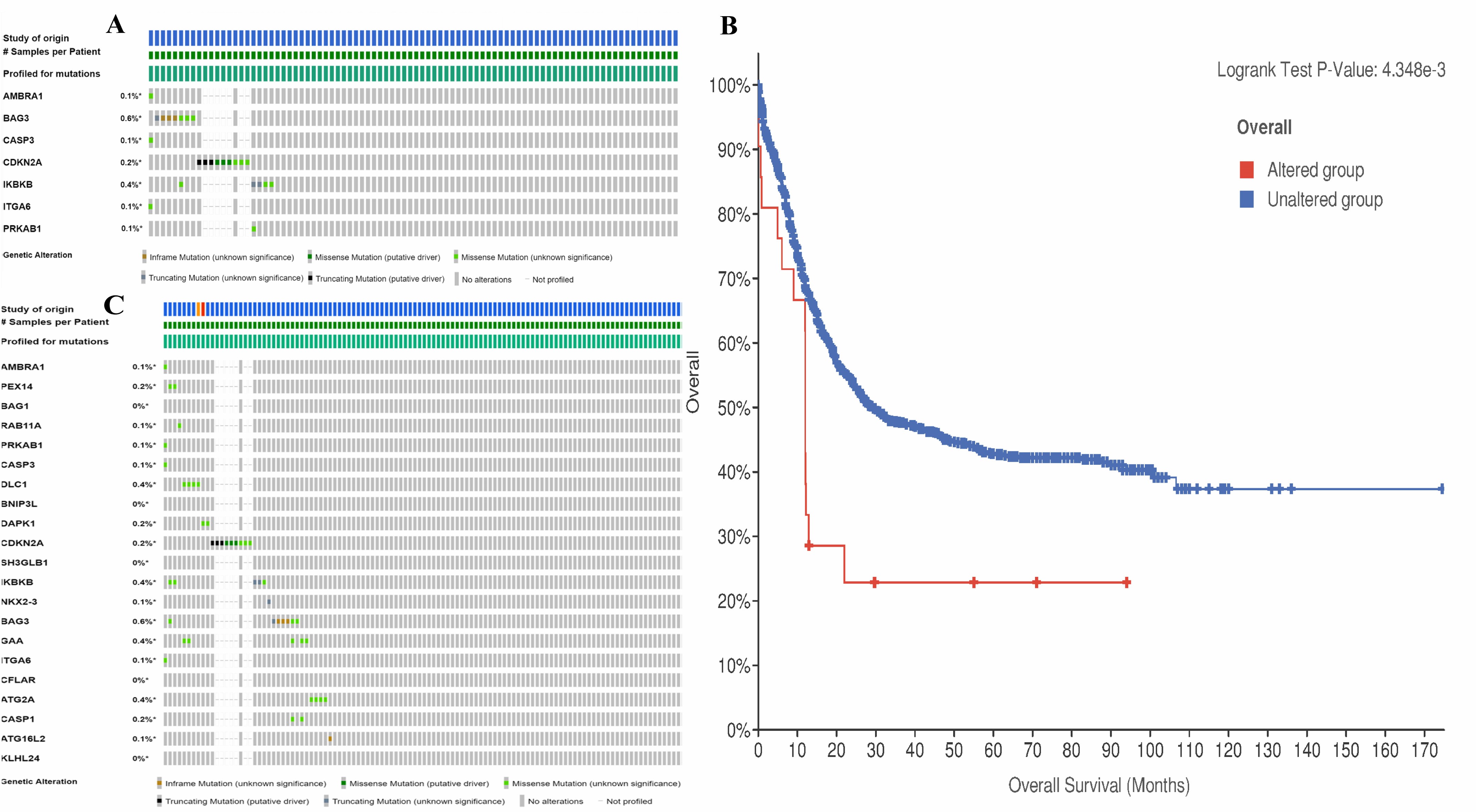

Supplement: Supplementary Figure 4 — Mutations in prognosis-related ARGs. This dataset was obtained from cBioportal for Cancer Genomics database, which containing 4,396 MDS samples. The mutation rate of 7 prognosis-related ARGs after multivariate analysis and 22 prognosis-related after univariate analysis were showed in (A, C). The survival analysis of 7 prognosis-related ARGs were showed in (B). [file Image_4.jpeg]
